# Assessment of knowledge, attitude and practice towards ante natal exercise among pregnant women attending antenatal care at Health centers of Mekelle, Tigray Region, Ethiopia, 2020

**DOI:** 10.1371/journal.pone.0262805

**Published:** 2022-08-09

**Authors:** Aregash Sitot, Haile Workye

**Affiliations:** 1 Department of Midwifery, College of Health science, Mekelle University, Mekele, Ethiopia; 2 Department of Nursing, College of Health science, Wolkite University, Wolkite, Ethiopia; Oregon State University, UNITED STATES

## Abstract

**Background:**

If pregnant mothers have no medical or obstetrical complications, they are encouraged to maintain active lifestyles during their pregnancies. Benefits of exercise during pregnancy include; prevention of gestational diabetes, pre-eclampsia and reduced low back pain. Therefore this study is done to assess knowledge, attitude and practice towards antenatal exercise among pregnant women who are attending antenatal care at health centers of Mekelle, Tigray Region, Ethiopia.

**Method:**

For this study cross-sectional study design was used. It was conducted from October 2019 up to January 2020 among 255 pregnant women who are attending antenatal care at selected health centers of Mekelle city. After the data were collected it was entered into epi-data 4.2.0 and was analyzed by using SPSS version 23.

**Result:**

Among the study participants of pregnant women 51%, 56% and 16.6% had good knowledge, positive attitude and practice towards antenatal exercise respectively. Among those 38.8%, 45.9% and 49.8% were expressed as antenatal exercise can decrease back pain, prevents excessive weight gain and increase energy and stamina during pregnancy respectively. Among those who practiced antenatal exercise, 95% and 83.4% was practicing with frequency ≥ three times per week and ≥20 minutes of the duration of exercise per session respectively.

**Discussion:**

Generally, this study showed that the level of knowledge, attitude and practice of pregnant mothers regarding antenatal exercise is poor. Therefore, health care providers who work on maternal health (Gynecologists, Midwives, Nurses, Health extension workers and other community workers) should provide counseling and health education on antenatal exercise.

## Introduction

Exercise is a physical activity that can be planned, structured and repeated body movement which is done to improve health and physical fitness. One of the health promotion and prevention of pregnancy-related complications is physical exercise during pregnancy [1].

Even if the type and the duration of antenatal exercise vary from person to person, studies showed that performing appropriate antenatal exercise has proven to be beneficial to risky mothers, though how much and what kind of exercise varies from person to person. Reports indicate that exercise during pregnancy can improve women’s psychological wellbeing, reduce gestational weight gain, back pain, length of labor, decrease cesarean section rates and reduce recovery duration. American congress of obstetrician and gynecologists (ACOG) recommended that participating in structured aerobic physical exercise during pregnancy reduce the risk of large weight for the new borne, rate of operative delivery, antenatal and postnatal depression and helps to cope up with labor pain and also antenatal exercise reduce risk of cesarean delivery by 15% than physically inactive [2]. Aerobic exercise at least 150 minutes per week is recommended for healthy pregnant and postpartum women [2,3].

Physical inactivity is the fourth leading risk factor of early maternal mortality worldwide [4]. It is estimated that due to physical inactivity pregnant mothers are at risk for breast and colon cancer (21–25%), diabetes (27%), ischemic heart disease (30%). Lack of exercise during pregnancy might result in loss of muscular and cardiovascular fitness, excessive maternal weight gain with a raised risk of Gestational Diabetes Mellitus, varicose veins, lower back pain, poor psychological adjustment [4,5]. A study done in Poland reported that there was a higher percentage of cesarean delivery (62%) in pregnant women who did not perform antenatal exercise than pregnant women who performed antenatal exercise (26%) [6].

Consequently, thousands of pregnant women pass away due to different risks which can be easily prevented by antenatal exercise. According to the Ethiopian demographic health survey(EDHS) 2016, in Ethiopia, there is high maternal mortality which is 412 death per 1000 women [7]. Although there is no global strategy that prescribes a list of interventions that will maximize and progress towards Ending Preventable Maternal Mortality (EPMM) in every country, to achieve the millennium development goals MDG5 in decreasing maternal mortality by two-third, by 2030 [8].

Regardless of the importance of physical exercise, some pregnant women and health care providers are concerned that regular physical exercise during pregnancy may cause fetal risks, but studies showed except vigorous physical activity under dehydrated conditions and exercise in the supine position, antenatal exercise is beneficial to pregnant women and fetus [9]. It is very important to create awareness about benefits and contraindication and engage in physical exercise to compact pregnancy-related complications. Moreover, it can give insight and baseline data for policymakers, the health care providers for future planning and emphasizing or developing antenatal physical exercise guidelines by integrating into maternity health service.

## Methods

### Study design and setting

Institution-based quantitative cross-sectional study design was conducted among 255 pregnant mothers at Mekelle city governmental health centers. Mekelle is the capital city of Tigray regional state. Administratively, the city is divided into seven sub-cities. It is located in the southeastern zone of the Tigray region and 781 km to the north of Addis Ababa, the capital city of Ethiopia. According to the 2010 population projection, the total population count of Mekelle town is 423,172. The town also possesses nine public health centers that provide antenatal services in Mekelle city. The study was conducted in selected public health institutions that provide antenatal services from October 2019 to January 2020.

### Study participants

All pregnant women attending ANC in selected governmental health facilities were randomly selected to be included in the study from October 2020 up to January 2021.

### Sampling method

The sample size was determined by using single population proportion formula. This study considered the proportions of pregnant that have a good attitude towards antenatal exercise that was conducted in kirkos sub-city, Addis Ababa which was 52.1%) [10] with 5% marginal error and 95% CI (α = 0.05).

By using this calculation formula, the sample size is 383. Since the number of source population is less than 10,000, then correction formula was used. Therefore, the final sample size was calculated to be 255.

Among the 9 public health centers which are found in Mekelle town that give ANC services 3 health centers were selected by simple random sampling technique.

Proportional allocation to each health facility was done by using the number of pregnant mothers who attend ANC in that governmental health institution based on the number of ANC attendant monthly reports before data collection. After that systematic sampling procedure was used to select ANC attendants. The sampling interval (K) was obtained by dividing the expected total number of monthly ANC service users 586(N) to the number of sample size 255 (n) at each data collection site. Every 2^nd^ pregnant mothers who fulfill the inclusion criteria was included until the required sample size is reached.

### Data collection

First the study was approved by Mekele University Health Science Research. Data were collected through face-to-face interviews by using a structured questionnaire. The training was given for the data collectors about the tools and ways of approaching to the study participants. The data were collected by five Midwiferies who were giving antenatal care. Before to interview, data collectors explained the aim and importance of the research for the study participants then written informed consent was taken from each study participant. The original questionnaire was prepared in English language then translated to the local language Tigrigna by the language expert for better understanding. To check the methods and materials the questionnaire was pre-tested before the actual data collection period. On each data collection days, the supervisors and investigators checked the completeness, accuracy, and consistency of the collected data in the whole period of data collection.

### Data analysis

After data collection, the questionnaire was checked for completeness and coded before data entry. Then the data was cleaned and entered into Epi-data 4.2.0. Then, the data was exported to SPSS and analyzed by using SPSS-version 23. Descriptive analysis was done for the variables. Finally, the results were presented using tables, figures and text accordingly.

### Operational definitions

**Good knowledge**: Participants answered correctly to16 knowledge questions, scored greater than and equal to the mean value, that recommended by ACOG.

**Poor knowledge**: Participants answered correctly to 16 knowledge questions and scored less than the mean value, that recommended by ACOG.

**Good attitude:** it refers to those who answered to 8-attitude question correctly and scored greater than and equal to the mean value, that recommended by ACOG.

**Poor attitude**: Those who answered to 8-attitude question correctly and scored less than the mean value, that recommended by ACOG.

**Good practice:** Those who exercise any type of antenatal exercise in frequency at least 3 times in a week and duration ≥20 minutes per session, that recommended by ACOG.

**Poor practice**: Exercise any type of antenatal exercise in frequency less than 3 times in week and duration<20 minutes per session, that recommended by ACOG.

## Results

### Socio-economic characteristics of study participant

The study included 255 pregnant mothers with a response rate of 100%. The age of the respondents ranged from 18 to 48 with a mean age of 26 years (SD = ± 5 years). Most respondents 101 (39.6%) were in the age groups 25–29 years. Out of 255 respondents who participated in this study, 244(95.7%) were married and 104 (40.8%) were a private workers. The majority of the respondent 237 (92.9%) were orthodox Christian. Most of the respondents 119 (46.7%) were secondary school. Two hundred seventy (85.7%) had an income level of ≥ 2000 ETB per month (**Table 1**).

**Table 1:** Socio-economic characteristics of pregnant women attending ANC at health center in Mekelle city, Tigray, Ethiopia, 2020(n = 255).

Variables	Frequency	Percent
**Age**		
15–19	9	3.5
20–24	83	32.5
25–29	101	39.6
30–34	50	19.6
≥35	12	4.7
**Religion**		
Orthodox	237	92.9
Muslim	14	5.5
Protestant	2	.8
Catholic	2	.8
**Marital status**		
Single	4	1.6
Married	244	95.7
Divorced	3	1.2
Widowed	4	1.6
**Occupational status**		
Housewife	49	19.2
Governmental employer	91	35.7
Private business	104	40.8
Non-governmental employer	11	4.3
**Educational status**		
Unable to read and write	9	3.5
Able to read and write	8	3.1
Primary school	24	9.4
Secondary school	119	46.7
Diploma and above	95	37.3
**Average monthly Income**		
<2000 ETB	29	11.4
≥2000ETB	226	88.6

### Knowledge about the benefit and contraindication of antenatal exercise

This study examined pregnant women’s knowledge about the benefit and contraindications of antenatal exercise. One hundred fifty six (61.2%) pregnant women ever heard about antenatal exercise. Out of the total respondents who heard about antenatal exercise 38.8%, 45.9% and 49.8% were expressed as antenatal exercise can decrease back pain, prevents excessive weight gain and increase energy and stamina during pregnancy respectively. However, 49.8%, 16.5% and 45.9%, of the respondents were correctly identified antenatal exercise helps to cope with labor and delivery pain, decrease the risk of gestational diabetic mellitus and high blood pressure during pregnancy respectively. Among the study participants around half of the respondents, 130(51%) had good knowledge about antenatal exercise (**Table 2**).

**Table 2:** Knowledge about the benefit and contra indication of antenatal exercise among pregnant women attending ANC in Mekelle health centers, Tigray, Ethiopia, 2020.

Characteristics frequency		Percent
Ever heard about antenatal exercises		
Yes	156	61.2
No	99	38.8
About what types of antenatal exercise had you heard?		
1. Walking	67	42.95
2. Aerobic	7	4.4
3. Relaxation or breathing	56	35.8
4. Pelvic floor exercise	8	5
5. Back care exercise	12	7.7
6. Ankle and toe exercise	6	3.8
Sources of information		
Health care provider	26	16.6
Family and friends	70	44.9
Mass media	53	34
Book	7	4.5
Antenatal exercise can Reduce back pain		
Yes	99	38.8
No	156	61.2
Antenatal exercise prevents excessive weight gain during pregnancy		
Yes	117	45.9
No	138	54.1
Exercise increases energy and stamina during pregnancy		
Yes	127	49.8
No	128	50.2
Exercise helps to cope with labor and delivery pain		
Yes	117	45.9
No	138	54.1
Reduces risk of gestational diabetes		
Yes	42	16.5
No	213	83.5
Decrease risk of high blood pressure during pregnancy		
Yes	117	45.9
No	138	54.1
Facilitate postnatal recovery time		
Yes	137	53.7
No	118	46.3
Prevents antenatal and postnatal depression		
Yes	15	5.9
No	240	94.1
Exercise benefits general health and development of the baby		
Yes	156	61.2
No	99	38.8
Exercise during Vaginal bleeding during pregnancy is contraindication		
Yes	150	58.8
No	105	41.2
**Contraindication exercise during** Uterine contractions		
Yes	187	73.3
No	68	26.7
Difficulty of breathing during pregnancy		
Yes	179	70.2
No	76	29.8
Chest pain during pregnancy		
Yes	158	62
No	97	38
Premature labor during pregnancy		
Yes	191	74.9
No	64	25.1
Poorly controlled type 1 Diabetic during pregnancy		
Yes	164	64.3
No	91	35.7
Dizziness during pregnancy		
Yes	135	52.9
No	120	47.1
Good Knowledgeable	130	51
Poor knowledgeable	125	49

### Attitude towards antenatal exercise among pregnant women attending ANC

The attitude assessment questions towards exercise during pregnancy among pregnant women show that about 68.6% of the respondents thought that physical exercise during pregnancy is necessary. Among the respondents 51.4% and 62.7% felt physical exercise during pregnancy is not risky to the fetus and helps to prevent pregnancy-related complications respectively. Among the respondents, 143 (56%) had a positive attitude towards antenatal exercise (**Table 3**).

**Table 3:** Attitude towards antenatal exercise among pregnant women attending ANC in Mekelle health centers Tigray Ethiopia, 2020.

Variables	Frequency	Percent
physical exercise during pregnancy is necessary		
**Yes**	175	68.6
**No**	80	31.4
Feels physical exercise during pregnancy is risky to the fetus		
**Yes**	124	48.6
**No**	131	51.4
Doing antenatal exercise suit with our culture		
Yes	131	51.4
No	124	48.6
Belief pregnant women should perform exercise under the guidance of health care professionals		
**Yes**	132	51.8
**No**	123	48.2
Feel exercise during pregnancy helps in post-delivery recovery		
**Yes**	154	58.8
**No**	101	41.2
Thinks antenatal exercise can reduce pregnancy-related complications		
**Yes**	160	62.7
**No**	95	37.3
Feel the exercising helps you get back your shape		
**Yes**	142	55.7
**No**	113	44.3
Feel exercise regimen should vary from one pregnant woman’s to another woman		
**Yes**	154	60.4
**No**	101	39.6
Attitude of ANE (summary index)		
Positive attitude	143	56
Negative attitude	112	44

### Practice of antenatal exercise among pregnant women attending ANC

Regarding the practice of antenatal exercise, this study indicated that 60(23.5%) of the respondents were practiced antenatal exercises in the current pregnancy. Among these who participated in the antenatal exercise, 60% practiced walking followed by aerobics 6(10%). Among those that practiced antenatal exercise, 58.3% and 83.4% were practicing with frequency ≥ three times per week and ≥ 20 minutes of the duration of exercise per session respectively. Moreover, the majority of them (38.4%) were guided by family and friends. Among the total participants, only 10% had Good level practice of antenatal exercise (**Table 4**).

**Table 4:** Practice of antenatal exercise during pregnancy among pregnant women in Mekelle health centers, Tigray Ethiopia, 2020.

Characteristics frequency		Percent
Practicing antenatal exercise in the present pregnancy (n = 255)		
**Yes**	60	23.5
**No**	195	76.5
Type of Antenatal exercise you exercised now (n = 60)*		
Walking	36	60
Aerobics	6	10
Breathing/relaxation	4	6.6
Pelvic floor exercise	4	6.6
Back care exercise	5	8.4
Ankle and toe	5	8.4
Advise to do ANC exercise(n = 60)		
Health care provider	18	30
Self	19	31.6
Family and friend	23	38.4
Frequency of exercise per week (n = 60)		
Less than or equal to 2 times	25	41.7
Greater than 2 times	35	58.3
Duration of exercise per minutes (n = 60)		
**<20 minutes**	10	16.6
**≥20 minutes**	50	83.4
Practice level		
Good practice	25	9.8
Poor practice	35	13.7

NB.

* denotes that multiple answer.

## Discussion

Antenatal exercise is tailored to promote health benefits to both pregnant women and fetuses. Despite this, exercises during pregnancy are poorly practiced in any parts of the world. It is better if pregnant women know the components, benefits, contraindications, and precautions to practice antenatal exercise effectively [11].

This study showed that the level of knowledge on antenatal exercise was 51%. This finding is similar to the study done in Nigeria among 189 pregnant women which is 51%. This similarity might be due to the use of similar methodology and comparable sample size [12].

The result of this study is higher than the study which is done in Zambia to assess the KAP of pregnant women on antenatal exercise which is 14% [13] and Colombo, Srilanka which is 16.3% [14]. The difference might be occurred because of different socioeconomic statuses and different study periods. Similarly, the result of this study also higher than the study which is done in Ethiopia, Addis Ababa on assessment of knowledge, attitude and practice towards antenatal exercise during pregnancy showed that the level of knowledge among the participants was 43.4% [11]. This difference could be due to the difference in availability of health care access and study period.

This study showed that among the respondents 56% had a good attitude towards antenatal exercise. The result is in line with a study that was done in India, which showed that the attitude of the respondents was 51%. This might be due to a similar methodology [15].

But this result is lower from the study which is conducted in Zambia on KAP of antenatal exercise among 279 pregnant women showed that 93% of respondents had a positive attitude towards exercise during pregnancy [13]. This difference might be occurred due to variations in social, cultural and economic status.

The result of the study also revealed that among the study participants who have good practice towards antenatal exercise was 10%. This finding is almost similar to the study conducted in Colombo, Srilanka among 110 pregnant women which showed that only 13.6% had good practice towards antenatal exercise [14]. This might be due to a similar methodology.

## Conclusion

Overall the results of the study reveal that the levels of good knowledge, positive attitude and good practice of pregnant mothers regarding antenatal exercise were 51%, 56% and 10% respectively. It showed that the level of good knowledge, positive attitude and good practice of pregnant mothers regarding antenatal exercise is poor. Therefore, health care providers who work in maternal health (Gynecologists, Midwives, Nurses, Health extension workers and other community workers) should put their significant effort to improve the knowledge, attitude and practice of pregnant mothers’ regarding antenatal exercise by providing better counseling and health education on antenatal exercise.

## Supporting information

S1 File (SAV)

## Abbreviations

ACOG: American congress of obstetrician and gynecologists
ANC: Ante Natal Care
ETB: Ethiopian Birr
EPMM: Ending Preventable Maternal Mortality
GDM: Gestational Dabitus Mellitus
MDG: millennium Development Goals
KAP: Knowledge Attitude and Practice
SD: standard deviation
PPS: Population Proportion to Size and SPSS: statistical package for social sciences
